# Narrow band quantitative and multivariate electroencephalogram analysis of peri-adolescent period

**DOI:** 10.1186/1471-2202-13-104

**Published:** 2012-08-24

**Authors:** EI Rodríguez Martinez, CI Barriga-Paulino, MI Zapata, C Chinchilla, AM López-Jiménez, CM Gómez

**Affiliations:** 1 Human Psychobiology Lab, Experimental Psychology Deparment, University of Sevilla, Seville, Spain; 2 Behavioral Methodology Lab, Experimental Psychology Deparment, University of Sevilla, Seville, Spain

**Keywords:** Spontaneous EEG, EEG development, Adolescence, Brain rhythms, Power spectrum, Principal component analysis, Component analysis

## Abstract

**Background:**

The peri-adolescent period is a crucial developmental moment of transition from childhood to emergent adulthood. The present report analyses the differences in Power Spectrum (PS) of the Electroencephalogram (EEG) between late childhood (24 children between 8 and 13 years old) and young adulthood (24 young adults between 18 and 23 years old).

**Results:**

The narrow band analysis of the Electroencephalogram was computed in the frequency range of 0–20 Hz. The analysis of mean and variance suggested that six frequency ranges presented a different rate of maturation at these ages, namely: low delta, delta-theta, low alpha, high alpha, low beta and high beta. For most of these bands the maturation seems to occur later in anterior sites than posterior sites. Correlational analysis showed a lower pattern of correlation between different frequencies in children than in young adults, suggesting a certain asynchrony in the maturation of different rhythms. The topographical analysis revealed similar topographies of the different rhythms in children and young adults. Principal Component Analysis (PCA) demonstrated the same internal structure for the Electroencephalogram of both age groups. Principal Component Analysis allowed to separate four subcomponents in the alpha range. All these subcomponents peaked at a lower frequency in children than in young adults.

**Conclusions:**

The present approaches complement and solve some of the incertitudes when the classical brain broad rhythm analysis is applied. Children have a higher absolute power than young adults for frequency ranges between 0-20 Hz, the correlation of Power Spectrum (PS) with age and the variance age comparison showed that there are six ranges of frequencies that can distinguish the level of EEG maturation in children and adults. The establishment of maturational order of different frequencies and its possible maturational interdependence would require a complete series including all the different ages.

## Background

The adolescent period is a crucial developmental moment of transition from childhood to emergent adulthood. It is also a period in which many different types of mental and behavioral problems can arise [1]. The EEG is able to provide information on two types of brain activity: spontaneous brain activity and event related potentials. This work focuses on the spontaneous brain activity during the peri-adolescent period. This continuous activity can be observed at any time and it is not apparently related to specific events. The spontaneous EEG activity is related to the neurofunctional states of the brain, in the normal, the pathological and the developmental subject.

The EEG is not stable over development, it is changing until arriving to the typical adult pattern. Several topics have been in the focus of the research investigating EEG development in control children: EEG power developmental trajectories [2-4], the establishment of developmental equations [5,6], microstates [7], brain complexity [8] and topographical changes during development [9,10], coherence developmental trajectories [11-13] and the relationship between the maturation of the EEG and neuroanatomical maturation [14]. The interpretation of the brain rhythms must take into account that brain rhythms are defined not only by the frequency and topography, but also for their brain sources and psychological reactivity [15,16].

### EEG power developmental trajectories, the establishment of developmental equations

The delta rhythm is the main activity in the first two years of life. In contrast, delta waves are not observed in normal adult EEG, in awake and relaxed states. However, the delta waves are characteristic of NREM sleeps stages III and IV also called Slow Wave Sleep in both adult and children [17]. The theta rhythm is mainly seen in children and decreases progressively with age, being characteristic of sleepiness and sleep [18], and it is enhanced in situations during the execution of tasks involving attention and working memory [19]. The visually modulated alpha rhythm, which is the main characteristic signature of the adult human EEG, presents a progressive maturation in which an increase of frequency occurs with increasing age [18] (see below). The beta rhythm has been classically linked to active information processing and arousal. Its amplitude increases until a period which includes the adolescence and the young adulthood [20].

The study of EEG power maturation during development is particularly important because EEG rhythms can be affected or modulated by developmental disorders [21,22], socioeconomic status [23,24] and gender [21,23,25,26].

With increasing age, lower frequencies decrease and higher frequencies increase [2,3,21]. A decrease in absolute delta, theta, alpha and beta band, a relative power decrease in the delta and theta bands and an increase of relative alpha and beta bands during the brain maturation appear. This result has been confirmed several times, and it seems to be an important landmark in EEG development [3,6,21,26]. This shift from low to high EEG frequencies is a characteristic signature of brain maturation.

The continuous or discontinuous nature of the developmental trajectory of EEG power is under a certain controversy, from a continuous linear change, as shown by the developmental equations [5,6] to a discontinuity marked by brain growth spurts at approximately 6, 10, and 14 years old [27,28]. Other parameters as the duration of microstates and the EEG coherence have also been linked to discontinuous developmental trajectories [7,29]. The presence or absence of cognitive abilities qualitatively different in different ages, as proposed by Jean Piaget [30], is behind the interest for this controversy. It has been suggested that the decrease in power of the slower EEG bands until the period of adolescence could be caused by synaptic pruning, typical of this period of development [14].

### Changes in frequency

Other important parameter changing during childhood is the frequency of brain rhythms. The frequency of the alpha rhythm is increasing during development. At the age of primary school, the alpha rhythm presents an average frequency of 10 Hz. This corresponds to the average frequency of the adult’s EEG. This value is achieved at the age of 10 [18], and this activity is found in the frontal, central and occipital areas. Recently, it has been proved that the increase in alpha frequency continues to 15 years old [31,32]. With increasing age (6 to 17 years old), Yordanova y Kolev [33] noted a decrease in slow alpha and a rapid increase of fast alpha in absolute power. In adulthood, this frequency is the most prominent in the EEG and shows the maximum amplitude in the parietal and occipital electrodes. Most of the developmental studies of spontaneous EEG have been performed using a broad-band approach. Cragg et al.[32], using a fine-grained approach, have confirmed that during adolescence there is a decrease in the amplitude of slow waves, an increase in fast rhythms and an increase in the frequency at which the peak of alpha is obtained. Alpha rhythm dominates when the subject is relaxed and more clearly in the eyes closed that in the eyes opened condition. It has been suggested that the increase in frequency during childhood in the brain rhythms would be related to the increase in speed of action potentials due to myelination and/or increase in axon diameter [10,20]. However, the latter proposal remains speculative.

### Changes in topographies

The presence of theta rhythm in posterior regions is common in children between 7 and 10 years old [9,10]. They suggest that this phenomenon may be a precursor of the alpha rhythm during maturation, overlapping areas involved in the generation of lower frequency alpha rhythm.

Shaw et al. [34] have pointed out that, in general, cortical maturation progresses in a posterior-to-anterior and lateral-to-medial fashion. A postero-anterior gradient of maturation has been described in the EEG [2,4,26,35]. For alpha and theta rhythms, and less marked in delta, maturation begins in the posterior and ends in the anterior regions. For the beta rhythm, maturation progresses from the center to the lateral and finally to the frontal regions [36]. The postero-anterior order of maturation for the human EEG rhythms would be related to the structural maturational trend.

### Relation to cognitive maturation

Some studies have shown that brain and cognitive maturation are intimately associated. For example, Hudspeth and Pribram [28] observed that through the development of cognitive functions during puberty, the brain maturation progresses from posterior regions to frontal regions. This postero-anterior pattern would be related to the fact that gray matter shows progressive maturation in a postero-anterior gradient [37].

Two of the basic developmental phenomena are related to myelination of axons and to synaptic pruning [37,38]. Between childhood and adolescence, gray and white matter are developing differently: gray matter decreases with age, possibly due to synaptic pruning [34,39], while white matter increases with age, possibly due to the myelination and increase in the diameter of axons [40]. This developmental pattern continues until the age of 20 years, and possibly continues during the third decade of life [1]. There is also, at least to a certain degree, regional variance in maturational processes, with different areas of the cortex maturing with different trajectories and at different times [41]. The anatomical regions in the frontal lobes seem to lose gray matter in a later period and are the last to mature, possibly producing the late maturing of cognitive processes [42]. The late functional and structural frontal maturation would be related to the late maturation of executive functions.

Furthermore, in studies of sleep EEG, Feinberg and Campbell [43] support the thesis of major brain reorganization during childhood and adolescence. They found that the maturational curves of three entirely different brain measures - synaptic density, cerebral metabolism and delta wave amplitude- show strikingly similar shapes with all three declining steeply across adolescence. They fitted the dynamics of these three parameters by a gamma function with a decay that prolongs in the third decade of life [44].

### Principal component analysis of the EEG

Principal Component Analysis (PCA) allows the multivariate EEG data be explained by a small number of latent variables (principal components). In previous reports, using the principal component analysis [45] allowed the separation of the fast components (alpha and beta) and slow (delta and theta) when the analysis was applied to children. Somsen et al. [3] were able to separate five bands: alpha, delta, theta, slow alpha and fast alpha. lmgren [46], Defayolle and Dinand [47] also described the components associated with classical EEG rhythms. Duffy et al. [48] obtained 20 factors including artifacts and the major brain rhythms in an adult population. Lazarev [49], using factor analysis of EEG of adults found four axes of variability related by the author with "general arousal", "activity in frontal areas," "cortical arousal" and "active inhibition selectively". The latter approach is not based strictly on the frequency bands, but includes other parameters defined by the author as the percentage of temporary presence of a given band, and the regularity of a band at the time. Tenke and Kayser [15] found five different components in the alpha range of adults, indicating that frequency PCA would be able to disentangle different sub-components in a certain frequency range. Barriga-Paulino et al. [10] have described that during maturation there is a component which presents an inverse relationship with delta and alpha. They also described that when the PCA analysis is applied to absolute power of the EEG, the first factor is related to individual variability of the EEG. The use of PCA on the narrow band frequencies of the EEG power would be able to extract subcomponents in some of the classical rhythms, which would show some topographical or frequency changes during the maturational process.

The main objective of this work is to study the EEG power pattern of maturation in human beings in the period from late childhood to peri-adolescent period, which are the periods surrounding the very important developmental period of adolescence.

Although adolescence is considered to be a period of major evolutionary changes at psychological and physiological levels, the EEG does not change significantly. At the age of 13, the teen shows an EEG similar to mature pattern [45]. Recently, it has been shown that during the adolescence period, absolute low frequencies power decreased, while high frequencies increased [32]. Also and inside this adolescence period (10–13 years old), the increase of alpha peak frequency continues. The novelty of the present approach is related to the systematic comparison between children and adults in the absolute PS by using a narrow band approach that would be able to reveal more subtle changes in the maturational patterns around the adolescence period, than those generally provided by broad band analysis of the classical brain rhythms. The narrow frequency band has been used to avoid scholarly biased grouping of frequencies. This is particularly important in developmental studies in which the frequencies of the latent structure of the EEG would be changing with age. In addition, the use of the Principal Component Analysis would allow to extract different subcomponents in the classical brain rhythms, particularly in the alpha band, in order to observe maturational changes associated to these sub-components. Although present study is not a longitudinal study but a cohort study in which the subjects were neurologically normal, and the analyzed records correspond to the differences between different subjects at different ages, the present experiment can still provide some useful information about neurophysiological changes associated to age.

## Methods

### Experimental procedure

#### Subjects

The study included a sample comprising 48 subjects (27 women and 21 men), aged between 8 and 23. Of the total, 45 were right-handed and 3 left-handed. The total sample was divided into two subgroups: a group of children and a group of young adults.

The group of children consisted of 24 subjects aged between 8 and 13 (mean age ± SD age: 10.1 years ± 1.41). Of these, 13 were females and 11 males (22 right-handed and 2 left-handed). The young adult group consisted of 24 subjects aged between 18 and 23 (mean ± SD age: 20.5 years ± 1.3). Of these, 14 were females and 10 males (23 right-handed and 1 left-handed).

Children and young adults did not report any neurological or psychological disease or impairment. Both groups were extracted from middle class socioeconomic background. Children were normal in academic records, and young adults were college students. Experiments were conducted with the informed and written consent of each participant (parents/tutors in the case of the children) following the Helsinki protocol.

### Electrophysiological recording

The EEGs were recorded during three minutes of spontaneous activity (which does not involve any explicit cognitive task) keeping the eyes open. The subjects were recorded at different times of the day, between 11 AM and 8 PM. No information about previous sleep was required. They were obtained from an average reference of 20 scalp sites of the International 10–20 system (Fp1, Fp2, F3, F4, F7, F8, Fz, FCz, T7, T8, C3, C4, Cz, P7, P8, P3, P4, Pz, O1, O2), using tin electrodes mounted in an electrode cap (EASYCAP, Herrsching-Breitbrunn, Germany) with two additional electrodes (M1, M2). Ocular movements (EOG) were recorded from two electrodes at the outer canthus of each eye for horizontal movements and one electrode under the left eye for vertical movements that were referred to FP1. All the scalp electrodes were re-referenced off line to the mastoid average (M1 + M2/2). Impedance was maintained below 10 Kilo-Ohms (KΩ). Data were recorded in Direct Current (DC) mode at 512 Hz, with a 20,000 amplification gain using a commercial Analog Digital (AD) acquisition and analysis board (ANT). Data were not filtered during registration. We asked the subjects to stay calm and looking at the screen for three minutes. Following to the three minutes of spontaneous EEG, the subjects were recorded in an ERP experiment which lasted for 20 minutes. The Event Related Potential (ERPs) results have already been reported [50,51]. The present results correspond to a narrow-band frequency analysis of the same data on spontaneous EEG that has been previously published using the broad-band approach, taken into account the classical delta, theta, alpha and beta EEG bands [10].

### Data analysis

A 0.1 Hz high-pass filter and a 20 Hz low-pass filter (zero-phase, low cutoff of 6 db/octave, high cutoff of 48 db/octave, BESA software) were applied to the data. The artefacts in the resulting EEG recordings were corrected by an artefact correction protocol. The algorithm used for the artefact correction was based on PCA (BESA software). This method splits the EEG components associated with cerebral activity from artefacts (ocular movements, muscular or cardiac activity), on the basis of spatial distribution, after which the EEG can be reconstructed free of artefacts [52].

After the correction of artefacts, an artefact rejection protocol was applied. All the epochs for which the EEG exceeded ±100 Microvolts (μV) in any channel were automatically discarded. The resulting records were reviewed manually and rejected those segments that appeared to be outside the parameters of brain activity.

The next step was to compute the Power Spectrum (PS) of the epochs by means of the Fast Fourier Transform (FFT). This tool consists of a mathematical function that transforms data that belongs to time domain into frequency domain [53]. Thus, it is possible to pass the EEG signal in time domain to its frequency spectrum and therefore graphically represent the spectrum of the electroencephalogram (in power or amplitude). The FFT algorithm implemented in the BESA software was applied to time domain data to convert them into the frequency domain using a cosine square window. The PS was computed in windows of 2 seconds (including 1024 sampling points given the 512 Hz), then all the segments averaged for each individual subject.

The analyzed time in the power spectral analysis were less than those initially recorded (90 epoch of 2 seconds) due to the elimination of epochs containing artifacts. The average time analyzed in the records of the group of children was 2'28'' (mean ± standard deviation: 2'28'' ± 0.41 minimum time 1'32'') while the average time recorded in the records of the group of young adults was 2'40'' (mean ± standard deviation: 2'40'' ± 0.45; minimum time: 2'20''). The frequency resolution was 0.51 Hz. Therefore, 39 frequencies (from the range 0–0.51 Hz to 19.38-19.89 Hz) were obtained, although the graphics have been rounded (20 Hz like extreme value). The EEG frequencies over 20 Hz were excluded from the analysis in order to reduce the impact of possible electromyography contamination of EEG recordings. Therefore, the original data matrix consisted of 960 rows (48 subjects x 20 electrodes) and 39 empirical variables in the matrix columns (39 frequencies).

For certain applications, the PS data were exported in selected pre-defined frequency bands for each subject: Delta (1 – 4 Hz), Theta (5 – 8 Hz), Alpha (9 – 12 Hz) and Beta (14 – 19 Hz) or selected based on the results. For certain applications, the PS averaged values for each band were collapsed by regions (anterior, central and posterior). For these particular exports, the electrodes that composed the anterior area were Fp1, Fp2, F7, F3, Fz, F4, F8; the central area was composed by FCz, T7, C3, Cz, C4, T8 and the posterior area by P7, P3, Pz, P4, P8, O1, O2. In these cases, the matrix for data analysis comprised 12 columns (4 bands x 3 regions) and 48 rows (subjects). For some applications, the same electrodes were collapsed but for different frequency ranges, which in the course of the analysis appeared as more suited for understanding the developmental issue.

### Statistical analysis

Using Statistical Package for the Social Sciences (SPSS) 14.0, a mixed-model **AN**alysis **O**f **VA**riance (ANOVA) was applied to the logarithm of the PS of absolute power to compare the children and young adults groups. The inter-subject factor was the variable age group with two levels: children and young adults. The within-subjects factors were the bands (four levels: delta, theta, alpha, beta) and the regions (3 levels: anterior, central, posterior). P values were computed using the Greenhouse-Geisser correction. The same type of ANOVA was performed to the six frequency ranges that in the course of the analysis appeared as more suited to the developmental issue (low delta, delta-theta, low alpha, high alpha, low beta and high beta frequencies).

In addition to the general ANOVA, the ratio of mean PS of adults with the mean PS of children was computed. The ratio of variances was also computed. This ratio was computed independently for each electrode and frequency and provides a landscape of the different frequency ranges in which different maturational trends can be obtained. Afterwards, and complementing the broad band ANOVA analysis, a narrow band mean comparison in terms of frequencies and spatial resolution was computed for the differences between children and young adults. T-tests were computed between children and young adults in each single electrode and frequency. For the mean comparisons of absolute power, a more detailed graphic with the different levels of statistical significance was also computed. No multiple corrections were applied to the t-tests because of the high statistical interdependence between the data, as proved by (i) the landscape of t-test across electrodes and frequencies, (ii) the high correlation between the PS of different frequencies and the small number of components explaining the variance of the whole population of data. The high internal dependence between the different empirical variables would potentially produce a huge number of false negative results if a conservative correction as Bonferroni was used. Anyway p-values in the t-test were very small, indicating big differences in the mean PS between children and young adults.

In addition to the narrow band between-subjects means comparisons, the between-subjects variance equality and correlation with age has been computed using F-Levene for variance comparisons and Spearman correlation coefficient respectively. These computations were obtained by each frequency and electrode. In addition to the statistical comparisons, we were interested in testing if there were different levels of maturation in different frequency ranges. For this purpose, the correlation with the EEG power in all electrodes and frequencies considered was computed. The topographical representation of this correlation was obtained in order to establish the level of maturation of different frequencies and scalp locations.

The mean and variance comparisons and Spearman correlations were computed in absolute and relative power. The relative power was obtained using Matlab 7.0 and using the following formula (eq. 1)

$$PRf_{i}=\left({PAf_{i}/\sum_{i=l}^{n=39}PAf_{i}}_{i}\right)100$$

PRfi: Relative Power for frequency i

PAfi: Absolute Power for frequency i

With all the previous analysis we expected to obtain an idea of the different maturational trends that would appear in different frequencies and electrodes.

### Cross-frequencies correlations

All frequency variables (39 frequencies) were correlated against the other in order to observe if there are patterns of covariation between different frequencies. This was done independently for the sample of children, for young adults and for the total sample. The two-tailed statistical significance of the correlations between different frequencies was estimated taking into account the number (N) of subjects (N = 24 for the children and young adults group, and N = 48 for the total sample). The correlation matrix was expressed in a color code to better appreciate the different cross-frequencies correlation patterns, and if they were different in children and in young adults.

### Principal component analysis

With this method, it is possible to identify the latent components that explain the variance of the experimental data [54]. In order to apply the PCA analysis, the original matrix was rearranged in 39 columns (from 0–0.51 Hz to 19.38-19.89 Hz). Therefore, the empirical variables introduced in the analysis were the different frequencies (39 variables) and the cases were the subjects by electrodes (480 = 20 electrodes x 24 subjects, for children and young adults groups, and 960 = 20 electrodes x 48 subjects’ cases for the total group). This rearrangement of electrodes is similar to that used by Tenke and Kayser [15], and it allows to have much more cases than empirical variables, as it is recommended for the PCA computation. Using SPSS 14.0, the PCA was applied to the different arrays. PCA was only applied to the absolute PS (data not normalized). PCA was computed without rotation and with Varimax rotation. The PCA was rotated by the Varimax method which maximizes finding a few components that accumulate most of the variance by maximizing the sum of the variances of the squared loadings. This method allows us to express the total variance of the data in a few components which can easily be identified as a source of variance in the empirical data. In this particular data set, it would allow to relate which components explain the different frequency bands variance. The explained variance of each extracted component was represented in order to evaluate how important was each extracted component in explaining the total data variance, and therefore, allowing to decide how many components are considered sufficient for explaining the empirical data (Scree Test). The total number of extracted components were 25, although only those indicated by the Scree test were considered for further analysis.

In order to establish the physiological meaning of each component, the loading factors of each empirical variable (the 39 frequencies considered) for the extracted components were represented in a color coded display. The loading factors are the correlation coefficient between a given empirical variable (PS for a given narrow band frequency) and the component scores of a given component. The two-tailed statistical significance of the loading factors was estimated taken into account the number of subjects (N = 24 for the children and young adults group, and N = 48 for the total sample). Component scores provide the value that a certain case (subject in a given electrode) has for a certain latent variable (the component). The correlation of the component scores with the subject age would give an indication on how a given component captures a maturational trend. The total number of component scores for each extracted component was 960 (48 subjects x 20 electrodes).

An important point was to find homologous components in the children, adults and total sample. To that end, two different methods were applied: the simultaneous representation of loading factors of the components versus different frequencies for both age groups, and the topographical representation of the component scores of the different components.

For the loading factors versus frequencies representations, these components which presented a similar profile were considered homologous across groups. In order to better observe if there was a similarity between components, the correlation between the loading factors of candidates to be homologous components was obtained. For the topographical maps of the component scores, those obtained by individual subjects were averaged independently for the children, young adults and total group, and represented by means of the EEGLAB topoplot function [55]. Additionally to the topographical map of component scores, the topographical map of the PS in the frequency at which the loading factor of a given component reached peak was displayed. Therefore, the strategy of identifying a given component was based on the frequency profile of loading factors and on the topographical maps of the component scores.

Specific questions that would be addressed with the PCA method and which are relevant to the understanding of the maturation of spontaneous brain rhythms are:

1) The comparison of the profile of the loading factors and the topographies of the different variables in children and young adults will assess whether the structure of the EEG is similar in both age groups or different.

2) If component scores of a certain component are inversely correlated with different frequencies, it would suggest an opposite maturational trend of certain frequencies during development.

3) By representing the loading factors vs. frequency of the homologous components of children and young adults, it would be possible to observe if the frequencies that are related to a certain component are shifted in frequency in late childhood as compared with young adulthood.

## Results

### Power spectrum

**Figure 1:**
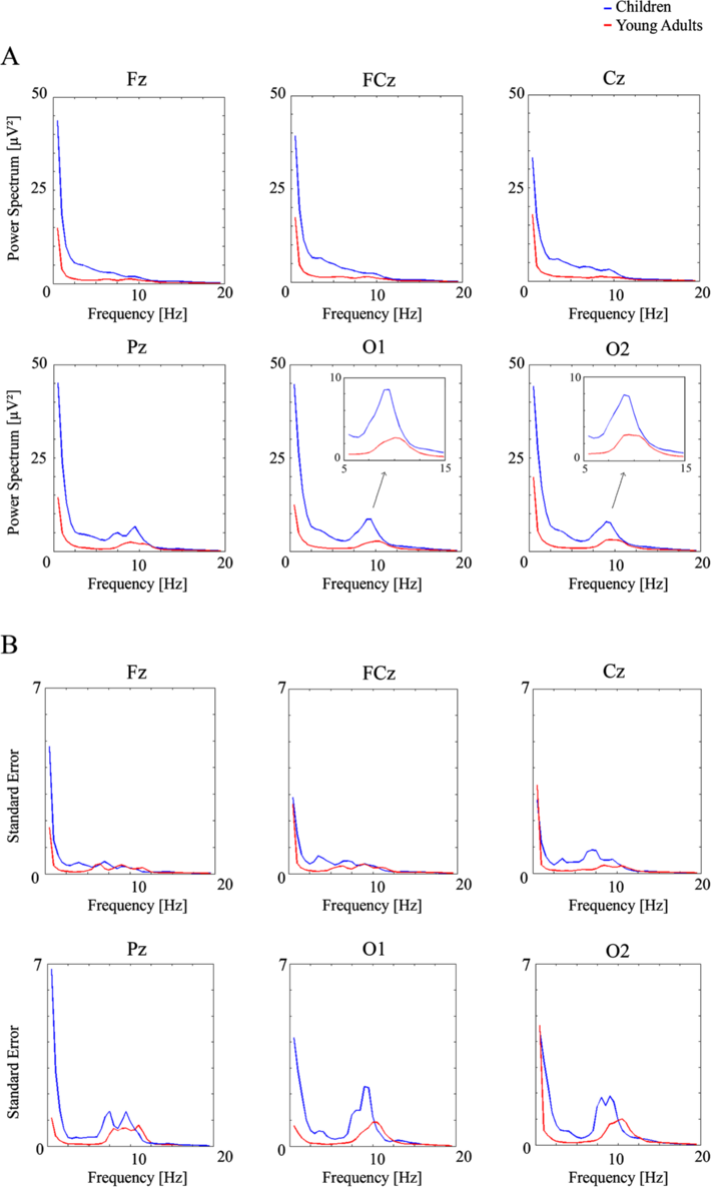
**Power spectrum (PS) (Figure **1**A) and the corresponding standard error (Figure **1**B) of children and young adults in electrodes Fz, FCz, Cz, Pz, O1 and O2.** Notice the higher PS in children than in adults. The insets in electrodes O1 and O2 show that the alpha peak is reached at lower frequencies in children than in young adults.

Figure 1 shows the average PS in absolute value in different electrodes in children and young adults. PS is higher in children than in young adults, and the differences are especially quite pronounced at low frequencies. At frequencies around 10 Hz, there is also a marked difference between young adults and children, but with a lesser magnitude than at lower frequencies. Also note that the alpha peak occurs in children at lower frequencies than in young adults (see also Table 1). The mixed ANOVA of the peak frequency in electrodes O1 and O2 as within-subjects factor, and age groups as between-subjects factor, showed that the group factor was statistically significant (F[1, 46) = 8.254), p < .006). The children reached the peak in lower frequencies than the young adults (measurements were done in the 7.5-12 Hz range).

**Table 1:** Peak frequency [Hz] of alpha in the scalp for the Electrodes O1 and O2

	***ELECTRODES***			
	**O1**		**O2**	
	**Mean**	**Standard deviation**	**Mean**	**Standard deviation**
*Children*	9.223	.926	9.350	.898
*Young*	10.094	1.345	10.200	1.258
*Adults*				

**Figure 2:**
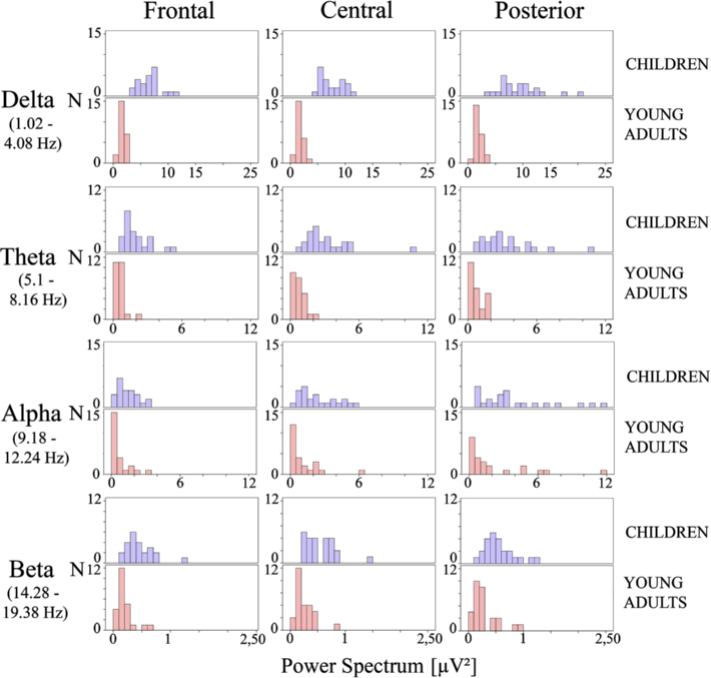
**Frequency histograms of the averaged PS values of frontal, central and posterior electrodes in the delta, theta, alpha and beta bands.** In all bands the children presented a higher mean than the young adults. Each histogram is created with 24 children or adults. The ANOVA statistics are referred in the results section. Averaging was obtained across subjects and electrodes as described in the methods section.

In Figure 2, the frequency histogram of the PS values in frontal, central and posterior electrodes (the electrodes were collapsed as indicated in the methods section) from the delta, theta, alpha and beta band are displayed. It can be observed that PS values are higher in children than in young adults in all frequency ranges and locations. The mixed ANOVA (age groups and scalp location factors) yielded statistically significant differences between children and young adults for each frequency band. For delta band, the group (F[1,46] = 267.32), p < .001), the scalp location (F[1. 909, 87.828] = 13.673, p < .001) and the group x scalp location were statistically significant ((F[1. 909, 87.828] = 8.524), p < .001) due to an increased PS in posterior sites. For the theta band, the group (F [1,46] = 65.126), p < .001, scalp location (F[1. 373, 63.167] = 25.993), p < .001 and the group x scalp location (F[1. 373, 63.167] = 7.674), p < .003 (due to an increased PS in central and posterior sites). were statistically significant. For the Alpha band, the group (F [1,46] = 14.204), p < .001, and the scalp location (F[1. 684, 77.459] = 112.813), p < .001 were statistically significant. The group x scalp location was not statistically significant. For beta band, the group (F [1,46] = 33.66), p < .001, and the scalp location (F[1.971, 90.687] = 7.361), p < .001 were statistically significant. The group x scalp location was not statistically significant.

**Figure 3:**
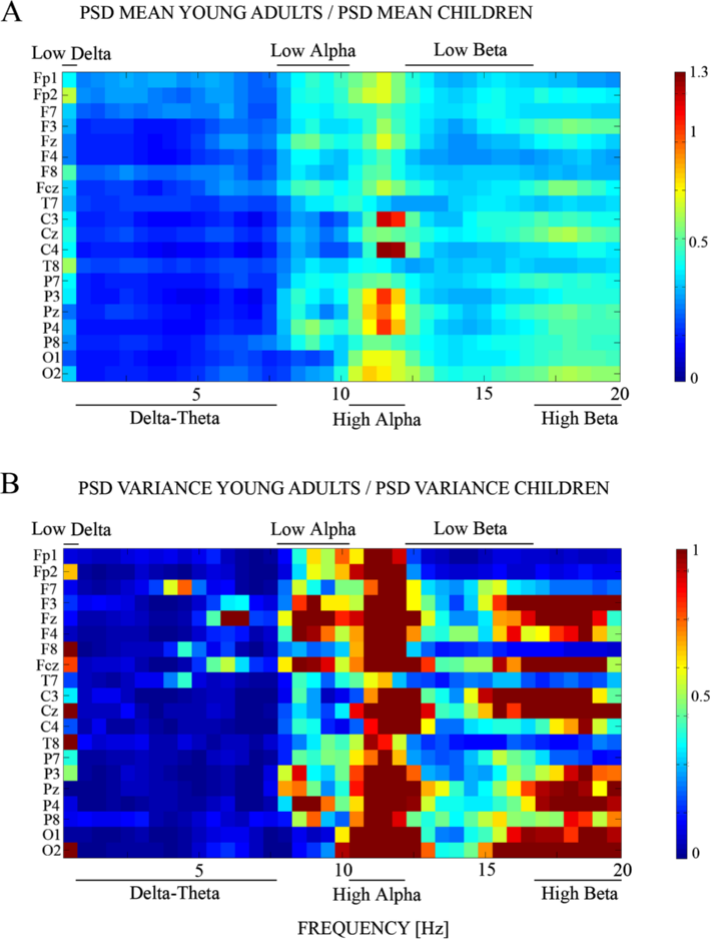
**Ratio of PS mean (3A) and variance (3B) of young adults and children.** Figure 3**A**. The figure shows the ratio of mean PS in young adults with PS in children (above). Figure 3**B** The ratios of variance are shown. In the case of variance, the color code has been saturated to 1.3. Notice the presence of six bands in which these parameters are relatively steady.

However, children should not have exactly the same rate of brain maturation in all the considered frequencies. For this reason, we computed for each electrode and frequency the ratio between the mean PS of young adults and children (Figure 3A) and the ratio of the variance of young adults and children (Figure 3B). The patterns of both, the mean and variance ratios seem to indicate that there are six frequency ranges in which these parameters seem to have a differential maturational rate: very low frequencies (0–0.51 Hz), delta-theta range (0.51-7.65 Hz), low alpha (7.65-10.2 Hz), high alpha (10.2-12.24, Hz), low beta (12.24-16.32 Hz) and high beta (16.32-19.89 Hz).

**Figure 4:**
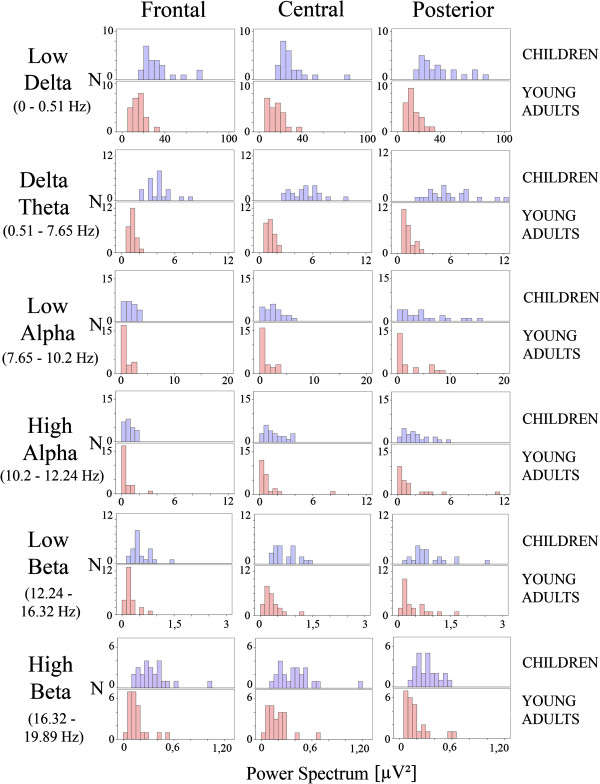
**Frequency histograms of the averaged PS values of frontal, central and posterior electrodes in the low-delta, delta-theta, low alpha, high alpha, low beta and beta bands, as suggested by Figure **3**.** In all bands the children presented a higher mean than the young adults. Each histogram is created with 24 children or adults. The ANOVA statistics are referred in the results section. Averaging was obtained across subjects and electrodes as described in the methods section.

The latter results suggest reorganizing the collapsing of frequencies for these new bands that seemed to reflect better the EEG bands maturational trends. Figure 4 shows the frequency histograms for these new bands. ANOVA were also recomputed for these bands. ANOVA of each new frequency band yielded statistically significant differences between children and young adults. For low delta frequency band only the effect of the group (F [1,46] =54.618), p < .001 was statistically significant. For delta-theta band group (F [1,46] =209.229), p < .001, scalp location (F[1.801, 82.852] = 18.378), p < .001 and group x scalp location (F[1.801, 82.852] = 9.598), p < .001 (due to an increased PS in central and posterior sites) were statistically significant. For low alpha band, only group (F [1,46] = 19.023), p < .001 and scalp location (F[1.459, 67.094] = 75.457), p < .001 were statistically significant. For the high alpha band, only group (F [1,46] = 11.316), p < .002 and scalp location (F[1.892, 87.050] = 119.735), p < .001 were statistically significant. For low beta band, group (F [1,46] =29.666), p < .001 and scalp location (F [1.984, 91.278] = 32.773), p < .001 were statistically significant. For high beta band, group (F [1,46] = 30.200), p < .001 and scalp location (F[1.929, 88.755] = 3.756), p < .029 were statistically significant.

**Figure 5:**
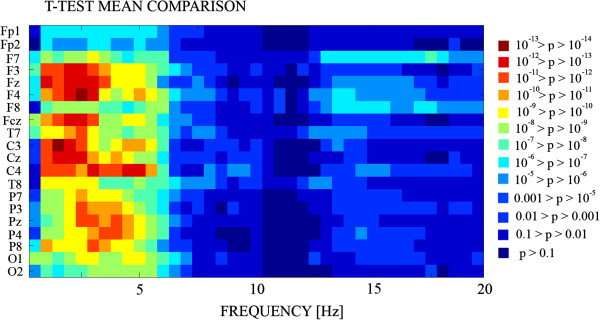
**T-test mean comparisons of PS of young adults and children.** Each pixel in the image represents the p-values for the *t*-test in a given electrode (rows) and frequency (columns). The highest statistically significant differences between children and young adults correspond to the delta-theta range. In any single case the mean PS of children was higher than PS in young adults.

**Figure 6:**
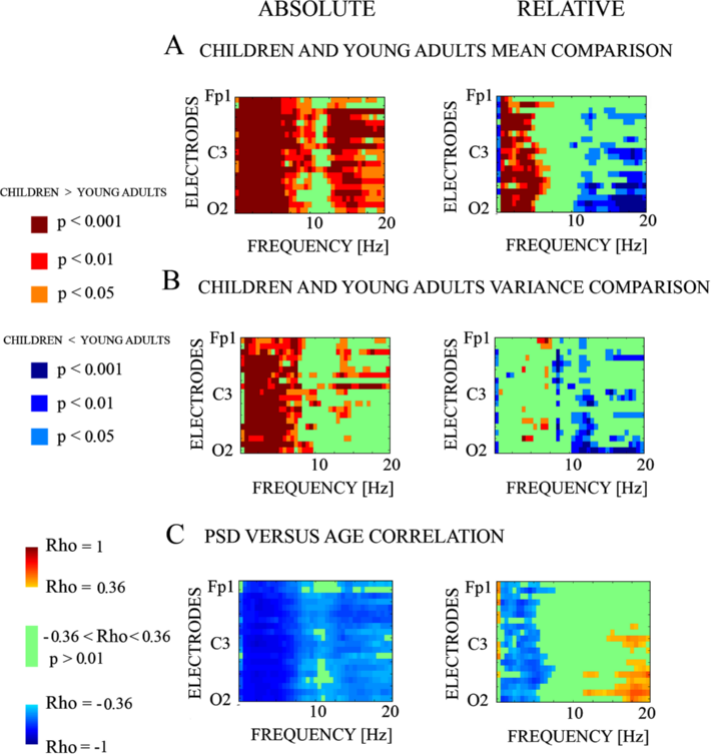
**Mean (Figure **6**A), Variance Comparisons (Figure **6**B) and Spearman correlations (Figure **6**C) for absolute and relative power.** The p-value of the *t*-test mean comparison of PS of children and adults (6**A**), the p-value of the F-Levene testing homogeneity of variance of children and young adults (6**B**), and the Spearman correlation of PS with age (6**C**) are displayed. Notice the differential pattern of absolute (left) and relative (right) power. Rho: Spearman correlation coefficient.

**Figure 7:**
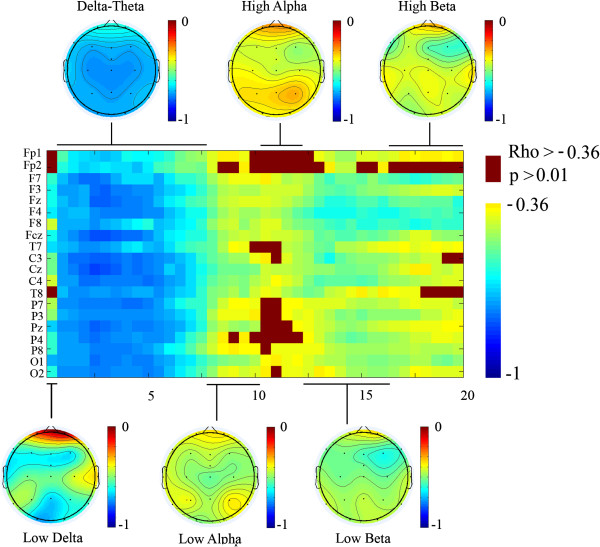
**Spearman correlation of PS of young adults and children with age.** Each pixel in the image represents the correlation coefficient in a given electrode (rows) and frequency (columns). The highest correlation coefficients correspond to the delta-theta range. The topographical maps of the mean of correlation coefficient for the frequency ranges indicated by lines are also displayed. Notice that correlation coefficient is more negative (better correlation with age) in anterior and central electrodes than in posterior electrodes for most frequencies. Rho: Spearman correlation coefficient.

However, the collapsing of electrodes in a few scalp regions and broad frequency bands could have missed certain patterns of maturational trends across frequencies and scalp locations. Therefore, we decided to make mean comparisons by means of t-tests (Figures 5 and 6), variance comparisons (Figure 6) and Spearman’s correlations with age (Figures 6 and 7), that would be able to reveal more subtle spatial and frequency patterns of maturation.

The level of signification for mean and variance comparisons and the correlation coefficient of PS vs. age would be indicators of differential trends of maturation of the different frequencies in different regions of the scalp. The signification of the t-test maps (Figure 5) confirms the existence of six frequency ranges in which the level of signification of t-tests between children and young adults are different, as suggested previously in Figures 3 and 4. With respect to the scalp locations, the t-test maps show a trend for higher statistical differences in the frontal and central electrodes than in posterior locations for the low delta, delta-theta and low and high beta ranges. The lowest levels of statistical signification were in low and high alpha bands.

Figure 6A shows the mean t-test comparison of young adults and children, the variance comparison (Figure 6B) and the Spearman correlations with age (Figure 6C). With respect to the mean comparisons, the main difference is the statistically significant higher PS in delta and theta in children for relative power, while the children present a reduction in the beta bands with respect to young adults also in relative power. The same pattern appears with the correlation with age, a negative correlation with age for the theta and delta band, and a positive correlation with beta. However, from a topographic point of view, and at least for beta, the mean and correlational effects are higher in posterior than in anterior sites. For variance comparisons, test F of Levene was also applied electrode by electrode and frequency by frequency. While the effects were in the delta-theta band for absolute power, the main variance statistically significant differences were in the beta band for relative power. These results indicate that any conclusion on spontaneous EEG maturation must take into account the type of transformation that has been computed on the PS data before statistical analysis.

In Figure 7, these differential statistical differences between anterior, central and posterior electrodes are further explored by means of the Spearman correlation between the PS values in each frequency and electrode with the subjects’ age. For the sake of comparison, the topographies of the correlations with age of the six proposed frequency bands are topographically represented. High correlation values between PS and age would be considered as an index of maturation from late childhood to young adulthood in both: frequencies and scalp locations. Following this criteria an order of maturation derived from this image would be that most anterior regions mature later than posterior sites. On the other hand those frequencies or locations with a low correlation coefficient with age would implicate an earlier maturation during childhood. With respect to the bands and following a criteria based on values of the correlation coefficient, but also in t-tests mean comparisons, a maturational order of high alpha, followed by low alpha and high beta, followed by low beta and very low delta, and finally delta-theta band can be proposed. Therefore, a certain trend of high frequencies maturing earlier than lower frequencies and posterior sites before anterior sites can be described*.*

An important point we would like to highlight in the analysis of PS in young adults and children is the comparison of results when absolute and relative power are compared, and when fractions of low frequency/high frequency PS values are considered.

**Figure 8:**
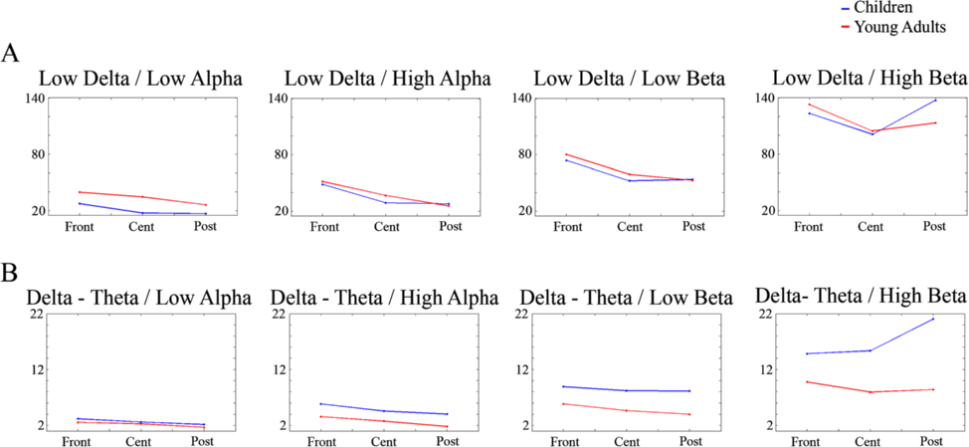
**Fractions of low frequencies/high frequencies in three different scalp locations.** The fractions of Low delta (8**A**) and Delta-Theta (**B**) Spectral Power are represented. Notice that the fractions of low delta did not have a difference between age groups, while those of delta-theta present a higher value in children than in adults indicating the dramatic decrease of delta-theta power in the adolescent period.

With regard to the comparisons low/high frequency, the comparisons between the low delta (Figure 8A) and delta-theta (Figure 8B) as numerators and low alpha, high alpha, low beta and high beta as denominators were computed. The ANOVA factors were the group age as between-subjects factor and scalp location as within-subjects factor. Only the group main effects and group x scalp locations interactions would be reported. In the low delta fractions and delta-theta/low alpha, there were no statistically significant main or interaction effects of the age group. For delta-theta/high alpha, there was statistically significant effect of the group (F [1,46] = 11.145, p < .002). For delta-theta/low beta, there was statistically significant effect of the group (F [1,46] = 18.425), p < .001). For delta-theta/high beta, there was statistically significant effect of the group (F[1,46] = 24.410, p < .001) and the group x scalp location interaction (F[1.873, 86.169] = 12,246, p < .001) due to an increased fraction in posterior sites. For all reported statistically significant cases, the fractions were always higher in children than in young adults (Figure 8).

### Correlational and principal component analysis

**Figure 9:**
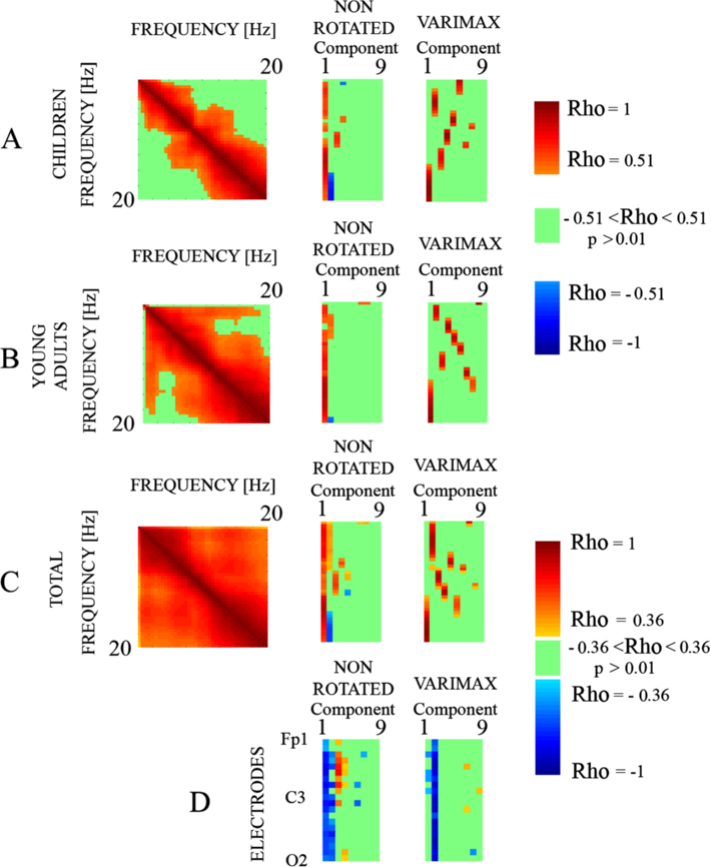
**A-C. Cross-frequency PS Spearman correlation (Rho) (left), loading factors of PCA analysis in the non-rotated PCA (middle) and rotated PCA (right).** In the left side of Figures 9**A**-**C**, it appears the cross-frequency correlation matrix for the children, young adults and the total sample. The Loading factors obtained from the non-rotated (middle) and varimax rotated (right) PCA are shown in the middle and the right of Figure 9A-C. **9D.** The Spearman correlation with age of the component scores was also computed (Figure 9**D**) for both non-rotated and rotated PCA.

The Spearman correlation matrix of 39 empirical variables (from 0 to 20 Hz) was computed. The correlation analysis performed with all frequencies tries to understand the internal structure of covariation of spontaneous EEG (Figure 9). The most notable results are that the highest correlations occur between close frequencies, all correlations being positive and generally lower in the children (Figure 9A, left) than in the group of young adults (Figure 9B left), suggesting that in the group of children the variability between different frequency bands is higher than in young adults. It would imply a certain asynchrony in the development of the different frequency bands. Finally, when the total sample is considered, all frequency bands provided a rich pattern of PS cross-frequency correlations.

**Figure 10:**
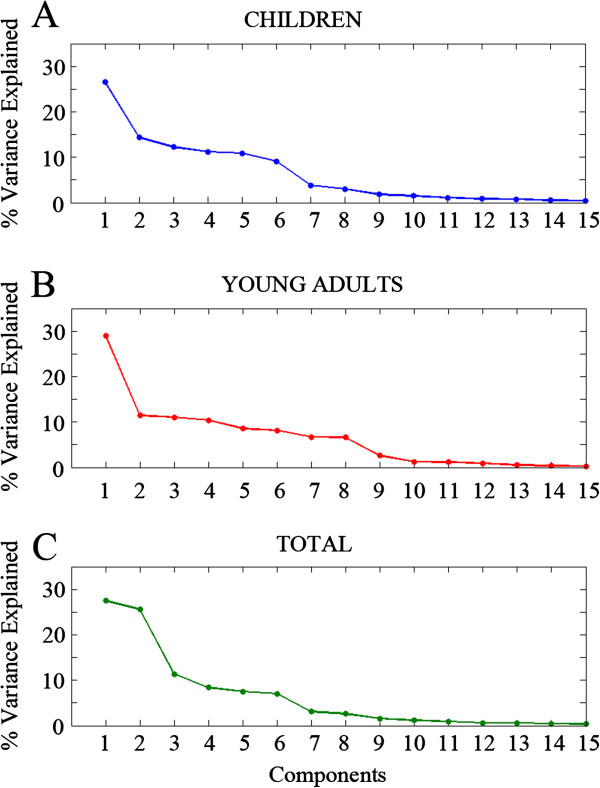
**Scree plot.** The image shows the explained variance by each of the components extracted in the Varimax-rotated PCA of PS data. The Scree plot is displayed for the children (10**A**), adults (10**B**) and total sample (10**C**).

Non-rotated and Varimax rotated PCA were computed in the matrix for 39 empirical variables (from 0 to 20 Hz). Figure 10 shows the Scree plot with percentage of explained variance by each component for Varimax rotated PCA, from which most of the analysis were derived in present report. There was a continuous change of slope that progressed until component number 9 in the case of young adults. Furthermore, as a physiological meaning was found to up component number 9, the 1–9 components (C1-C9) were taken into account for exploring some aspects of the latent structure of the maturation of human EEG.

Loading factors of the components for non-rotated and rotated PCA are shown in the middle and right side of Figure 9A, B and C. Loading factors represent the correlations between empirical variables (the EEG frequencies) and component scores of the different extracted components. The rotated loading factors point out to a better segregation of different frequencies in the rotated PCA than in the non-rotated PCA, suggesting that rotated components capture the variability due to the basic brain rhythms present in the spontaneous EEG. However, the non-rotated PCA is also mathematically valid and can give insight on some aspects.

Loading factors of the first component of the non-rotated PCA incorporate the individual variability, as it is shown by the high correlation with all the frequencies, indicating an individual variability meaning for this factor. Interestingly, the second component shows a reverse pattern between delta and alpha frequencies, but only for the total sample, indicating that during development, a latent factor should be acting with opposite effects in these two frequency ranges. For Varimax rotated PCA, the most interesting aspect is the excellent segregation of different frequency bands (Figure 9, right) which roughly corresponds to the 6 bands segregated in the previous PS analysis of mean, variance and correlation comparisons described in the previous PS section.

Correlation with age of component scores was also computed (Figure 9D). The general landscape suggests a better correlation with age in the anterior electrodes (upper part of the Figure 9D) than in the posterior electrodes. More specifically, the first three components of non-rotated and the two first of rotated components are those which present the better correlation with age, and for that reason would be the components which would mature in more advanced ages. The correlation with age can also be considered as a size effect of the ANOVAs of component scores between the two age groups which are later described in Table 2.

**Table 2:** Statistical comparison of the component scores of children and young adults when PCA is computed from all subjects

	**Levene test:**	**Mean comparison:**
C1 (Beta - EMG)	(F[1, 958] = 28.936), p < .001	(F[1, 705.346] = 6.488), p < .001
C2 (Delta - Theta)	(F[1, 958] = 361.333), p < .001	(F[1, 561.066] = 32.359), p < .001
C3 (Alpha)	(F[1, 958] = 48.738), p < .001	(F[1, 695.931] = 2.455), p < .014
C4 (Mu)	(F[1, 958] = 6.373), p < .012	(F[1, 691.765] = −1.610), p < .108
C5 (Low Alpha)	(F[1, 958] = 117.238), p < .001	(F[1, 522.495] = 2.954), p < .003
C6 (High Alpha)	(F[1, 958] = 72.075), p < .001	(F[1, 741.052] = .975), p < .330
C8 (Low Delta)	(F[1, 958] = 76.309), p < .001	(F[1, 728.777] = 4.176), p < .001
C9 (Mu)	(F[1, 958] = 15.962), p < .001	(F[1, 917.456] = 1.842), p < .066

The F values of Levene test (homogeneity of variances) and One-way ANOVA (two between-subjects levels: children and young adults) are displayed. The rhythms associated to each component are indicated from component 1 (C1) to component 9 (C9) . In the case of C1 the term Beta-EMG refers to the possible contamination of the Beta rhythm by electromyographic signals.

In order to identify the physiological meaning and the homology of Varimax components in the two age groups, two different strategies were followed: the representation of loading factor vs. frequency, and the topographical maps of component scores. Two components would be considered homologous if they share similar patterns in both. In the case that two components are homologous, it would also indicate that the general structure, in terms of topography and frequency of a given rhythm, is already mature in late childhood.

**Figure 11:**
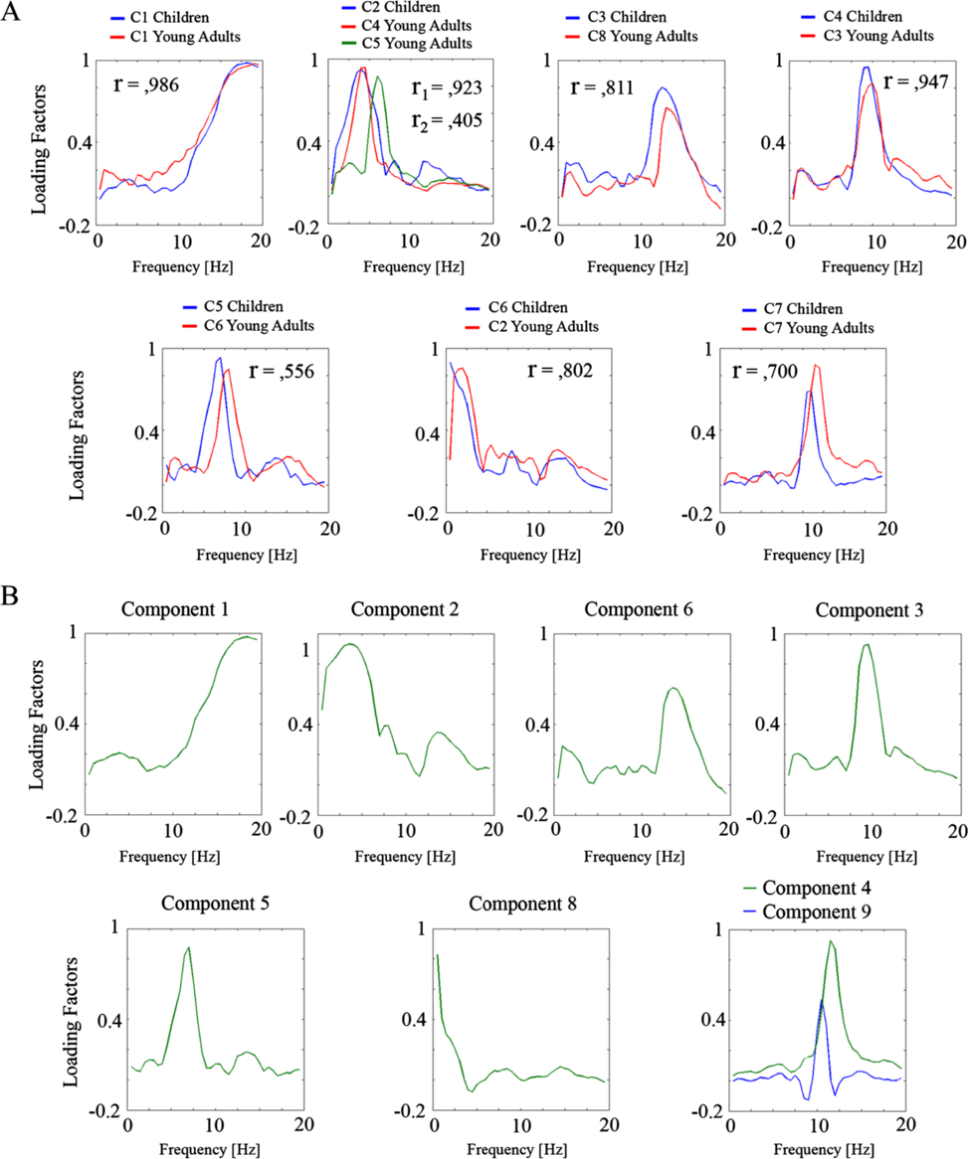
**Loading factors versus frequency.** 11**A**. The values of the loading factors vs. EEG frequency of the components in which similar patterns in children and young adults were obtained are represented. The Pearson correlation coefficient (r) computed comparing the loading factors of children and young adults are also represented. Notice that in most cases the r value is relatively high. For the C2 of children, two components of young adults were considered homologous due to the similar pattern of the loading factors, but also because similarities in the topographies of the component scores (see Figure 12). 11**B**. The values of the loading factors versus EEG frequency of the components obtained from the total sample. Components 4 and 9 seem to explain the same range of frequencies and present similar topographies (see Figure 12).

Varimax rotated loading factors vs. frequency appear in Figure 11A. Those components in which the relationship of loading factors with frequencies is similar in children and young adults have been represented overimposed. As shown in Figure 11, the patterns are relatively similar in both groups. Loading factors of the different components appear to have peaked in beta, delta-theta, high alpha, alpha, low alpha, low delta and possibly mu rhythm (between 10,71 - 11,73 Hz). For the case of delta-theta, two different subcomponents appeared in this range for young adults, although the component representing high theta had a low peak loading factor value. Note that for most cases, loading factor peaks in children occur at lower frequencies than in young adults. The figure also shows the correlations between loading factors of children and young adults, which were high in most cases indicating a similar profile in children and young adults. Figure 11B shows Varimax rotated loading factors extracted from total sample versus frequency. A similar pattern to that of children and young adults is obtained, indicating that the same type of rhythms is present in the groups of young children and adults. A complete characterization of the physiological meaning of components requires the analysis of component scores topographies.

**Figure 12:**
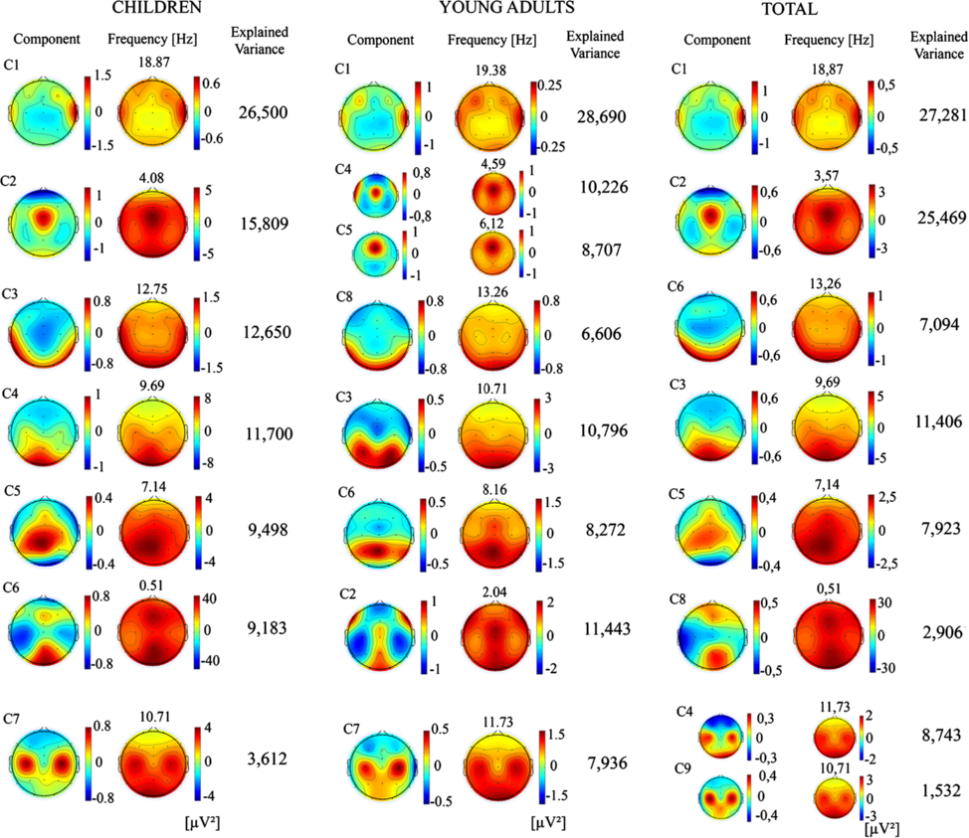
**Topographical representation of PS and component scores of topographies of each component.** Topographies of component scores of children, young adults and the total sample are displayed. For the PS map, the frequency in which the loading factor of the component peaked (see Figure 11) was also represented. The explained variance is also indicated. The cases in which the component scores have very similar topographies (and profiles of loading factors vs. frequency are also similar, Figure 11) are plotted together and considered as homologous components between children and young adults.

In Figure 12, PS topographies of each component and component scores topographies of the components of children, young adults and the total sample are displayed. For the PS map, the frequency in which the loading factor of the component peaked was represented. The explained variance corresponding to each of the components is also represented. Topographies of PS and loading factors are compatible with topographies of beta, theta (and anterior delta), high alpha (occipito-temporal), occipital alpha, low alpha (parieto-occipital), low delta and possibly mu rhythm. It must be noticed that similar topographies of PS and component scores components maps for the children, the young adults and the total sample group were obtained, indicating a similar latent structure of the brain rhythms around the peri-adolescent period.

Table 2 shows variance and mean comparisons of component scores of different components obtained in the total sample. As components represent individual rhythms, the lack of statistical differences in mean comparisons of component scores would indicate an early maturation of these rhythms, while the statistically significant differences would indicate a delayed maturation. The components that did not show statistically significant differences in mean comparisons were components explaining the high alpha (C6) and mu rhythm (C4 and C9). Levene test for different components showed that all variances of component scores were statistically significantly different between both age groups.

One last interesting suggestion from the analysis of loading factors (Figure 11A) and components scores (Figure 12) is the fact that different subcomponents in alpha range seem to peak at lower frequencies than young adults.

## Discussion

Present results showed that the well described pattern of decrease in EEG power with age would be organized in six different frequency ranges. The correlation pattern of different frequencies suggests a certain asynchrony in the maturation, which would be a consequence of the six frequency ranges with different EEG power ratios between children and adults in the peri-adolescent period. The narrow-band frequency correlation with age analysis corroborated the previously described pattern of an earlier maturation of posterior regions with respect to anterior regions. But low frequencies present a lower rate of maturation in the peri-adolescent period than alpha and beta rhythms when criteria based in mean comparisons and correlations comparisons are used. However, the delta-theta/high frequencies ratios were decreasing with age. The principal component analysis allowed extracting the following basic brain rhythms in children and adults with very similar topographies: beta, theta (and anterior delta), high alpha (occipito-temporal), occipital alpha, low alpha (parieto-occipital), low delta and the mu rhythm. The scalp alpha rhythm and all the extracted alpha sub-components and the extracted mu rhythm peaked at a higher frequency in young adults with respect to children. Given the different pattern of age differences when absolute and relative power are compared, some caution must be taken when extrapolating conclusions from one to the other type of analysis.

### EEG power differences around the adolescence period

Children showed greater absolute spectral power than young adults in the four standard frequency bands. Spectral power decrease during maturation is a general finding [2-4,20,21,32,56]. This decrease would be attributed to a decrease in the number of cortical synapses due to synaptic pruning [14,43,44]. In addition, the reported metabolic decrease with the age obtained with PET extends throughout adolescence, and may be associated with this synaptic pruning [57]. The possibility that a part of this decrease would be due to a lower electrical resistance of pericranial tissues (meninges and skull) in children compared to adults cannot be ruled out. However, magnetoencephalography (MEG) recordings have shown that also in MEG theta activity has greater amplitude in children than in adults [9]. Given that the skull has a high magnetic permeability, the MEG decrease in theta with age must necessarily be attributed to a current decrease in brain generators during maturation. Hagemann et al. [58] have shown, in adults, a minor contribution of skull thickness variability to the alpha EEG power variability. Therefore, at least in part, increased EEG amplitude in children is probably due to brain rhythms generated in more intense amplitude than in young adults, probably due to an increased number of synapses in children with respect to young adults.

The obtained differences in spectral power in present report were higher in the delta and theta rhythms, as it has been previously described [3,58]. Looking at the obtained data more carefully, when the ratios of mean and variance EEG power and the correlations with age of EEG power are considered, six different frequency ranges with a different rate of maturation are obtained. Those ranges correspond to low delta, delta-theta, low alpha, high alpha, low-beta and high-beta. It implicates that the rate of EEG power maturation is different for different frequency ranges at the peri-adolescent period. Ideally, this result would be able to provide an order of maturation for frequencies in the 0–20 Hz range. However, it is not possible to assume that EEG maturation is complete in this group of young adults, and for instance, the apparent higher maturation in the high alpha range with respect to the other frequencies would be optionally due to a lack of maturation in the young adults, instead of a complete maturation in adolescents. A complete answer to this question would need the establishment of an age complete sequence of EEG maturation covering the third decade of life.

However, in the case that EEG power maturation is already almost completed in our 18–23 years old sample, the following order of maturation for EEG absolute power frequencies can be proposed: high alpha, followed by low alpha and high beta, the low beta and low delta and finally the delta-theta range. This possible order of maturation is different to the general view of low frequencies maturing earlier than high frequencies [2,4]. A similar decrease of delta until young adulthood has been recently observed [58]. Part of this controversy must be due to the fact that the arguments about increasing of high frequencies with age refer to relative power [5]or to fractions [4,21], but also to different ages in different studies [21,32]. The relative power has the problem of an artificial increase of high frequencies due to the dramatic decrease of lower frequencies. For this reason, the fractional method seems to be more suited to address the problem of the contributions of each frequency band to the whole spectrum. Looking at the absolute decrease or increase of absolute EEG power, it should be considered the major descriptor of EEG maturation. In fact, in present results, the delta-theta/high frequencies fractions were statistically significantly higher in children than in young adults. The latter result is due to a higher decrease of delta-theta than high frequencies with age, corroborating the higher rate of maturation of delta-theta during adolescence with respect to alpha and beta. A similar result has been recently obtained by Lüchinger et al. [55] for the 0–20 Hz frequency range, although they found a continuing maturation of beta in frequencies higher than the 20 Hz limit used in the present study. The present proposal of maturation order, around the adolescent period, is based on the comparison of the amplitudes of the different frequency ranges, and it can be distorted, particularly in alpha, by the fact of a different frequency for the same rhythm at different ages. The extraction of PCA components would help in solving if there is an earlier maturation of alpha with respect to delta-theta.

Interestingly, when the cross-frequencies EEG power correlations are obtained, the young adults obtained a higher pattern of correlation than the children. The latter result and the previously described six frequency ranges differential maturation indicate that there is possibly a certain asynchrony in the maturation of the different rhythms. Somsen et al. [3] showed differences in maturational rate of different rhythms in children between 6–11 years old. Also an asynchrony pattern of EEG development between different areas of the brain was noticed by [28] in late childhood. The developmental trajectories of non-REM delta and theta during sleep have also shown different developmental trajectories: delta presenting a plateau until 10 years old and then decay, while theta presents a continuous decay [59]. Gasser et al. [4] also noticed a decrease in absolute EEG power with age (6–17 years old) except for high alpha, but the dynamics of EEG decrease in most bands were not parallel. The fact that in young adults the correlations are relative high suggests that, in this group of age, the dominant factor is the individual variability in EEG power, which affects to 0–20 Hz EEG frequencies. Another interesting point arising from correlation matrices is that absolute power frequencies are highly correlated with close frequencies. This is probably due to what is already seen in power spectra, where there are smooth transitions between spectral powers of different frequencies.

Mention apart requires the obtained result that the EEG power maturation in the range of very low delta (range 0 to 0.51 Hz) seems to be different than in delta-theta frequency range (.51-7.65 Hz). However, it must be kept in mind that this frequency is within the range of the high pass filter located at 0.1 Hz and 6db per octave frequency cutoff. Nevertheless, records in young adults and children received the same type of filtering, and therefore, it is difficult to assume that this difference is a consequence of signal processing. Tenke and Kayser [15] found a 0.8 Hz component with an anterior topography that they considered eye movements’ artifact. However, the low delta component described in present report has a lower frequency, it is posterior and our recordings were artifact-corrected. For these reasons, it is highly improbable that the low delta component would be considered exclusively an eye-movements artifact in present report. Pfurtscheller [60] described a 0.1 Hz brain rhythm. This low delta rhythm demonstrated to modulate the amplitude of the alpha and delta rhythm [61]. The differential maturation rate of the low delta rhythm obtained in present report points out to an independent low delta rhythm from a faster delta rhythm, more associated to the maturation of theta. However, at this point, it is necessary to be cautious and new records and analysis are needed, particularly given the technical difficulty of obtaining a true DC recording.

The topographical pattern of the correlation of PS with age shows that, in general, correlation is higher in anterior than in posterior sites for the frequencies considered in present report, suggesting that maturation is progressing from posterior to anterior sites. The correlation with age pattern is quite similar to the mean PS comparison between age groups. In this sense, the correlation with age pattern can be considered as a measure of the size effect of mean comparisons. The present results extend to a narrowband PS analysis, previous results on broad band showing that maturation in general progresses from posterior to anterior sites. Hudspeth and Pribram [28] observed that the latest EEG maturation of EEG power occurred in frontal sites. These results would be coherent with the antero-posterior maturational rate obtained in present report. Otero [62] indicates that for theta and alpha rhythms, and to a lesser extent delta, maturation begins in posterior regions and ends in anterior regions. For beta, maturation progresses from central to lateral and finally to frontal regions. Our results suggest that the anterior-posterior rhythms pattern of maturation is general for all the frequencies considered, but with certain patterns of regional differences. This antero-posterior pattern of maturation would be related to progressive increase of frontally related cognitive functions. In this sense, several studies have shown that EEG and cognitive maturation are intimately associated [7,29]. Hudspeth and Pribram [28] observed that EEG maturation progresses from posterior to frontal regions, possibly permitting the increase of more complex cognitive functions as age progresses. Recently, it has been consistently proved that brain maturation development shows progressive (axon myelinization) and regressive (synaptic pruning) phenomena [37,38]. More specifically, grey matter decreases with age possibly due to synaptic pruning [34,39], while white matter increases with age probably due to axon myelinization and/or axon diameter increase [1,40]. This pattern of inverse trajectories between grey and white matter continues until 20 years old [1]. An interesting result, which is possibly underlying the late PS maturation of anterior sites obtained in present report and others, is that frontal lobes seem to lose grey matter at a later period than posterior locations, so they are the last to mature [14,42].

In this section of differences in PS between children and young adults, the different pattern of maturation when absolute and normalized power is used must be remarked. The normalized PS measures have been used to minimize the impact of the strong individual component in PS amplitude values and to increase test-retest reliability of EEG measurements [5], but this normalization procedure could produce some direct questions about their direct extrapolation to the values obtained in the absolute PS. Particularly a high interdependency between these measures can be obtained as a by-product of the normalization procedure [63].

The results obtained in present report show the characteristic higher normalized PS of low frequencies in children over young adults, and the higher PS in beta of young adults with respect to children. Correlation with age showed a similar pattern. This result has been classically obtained, showing the typical movement from slow to fast waves with age [2-4,45], indicating that the normalized power is more sensitive to changes in composition of the frequencies with age than absolute frequencies [21]. However, some topographical differences are obtained when statistically comparing the mean, variance and correlation with age between absolute and normalized power. Particularly, differences are more accused in anterior beta in absolute power, while differences are more marked in posterior beta when normalized power is used. Gasser et al. [4] also found an increased relative power in centro-parietal (beta) and parieto-occipital (high alpha) with age. This different result in normalized (relative) power with respect to absolute power would be a consequence of the normalization transformation, and suggests some caution when absolute and normalized power are considered.

### Principal component analysis

The purpose of using a PCA analysis would be to find different sub-components in the brain rhythms which are not obvious in the scalp EEG, and demonstrate if they show some maturational trends. In this approach, we used Varimax rotated and non-rotated approaches. Both approaches are mathematically valid, the rotated Varimax approach tries to accommodate the data variance in a few components in order to simplify the physiological interpretation, and for this reason, we have chosen this approach for the interpretation of the results, except for the results obtained in the non-rotated PCA in the first component and in the third component.

The loading factors of the first component of non-rotated PCA were very high and the component scores were correlated with age. This first component of non-rotated PCA could be related to the "general alert" factor described by Lazarev [49] in adults and in children by [3]. Wackermann and Matousek [64] described a non-linear age factor which accounted for most of the EEG power variance. In a previously reported analysis [10], applied PCA to the absolute broad-band PS showed that the first component is strongly associated with the average energy or average power spectrum. Therefore, this first component could be associated to the individual variability EEG power which is therefore decreasing with age. The second component presented the interesting characteristic that the loading factors were inverted in sign with the delta and the alpha band, suggesting a partial opposite inter-dependency from a single factor of the maturation of these two bands. The latter result was also previously described in the broad band analysis of this data [10].

The pattern of loading factors in rotated components, in terms of loading factors vs. frequency and topographies of component scores, would allow to characterize the physiological meaning of different components and to prove if children and young adults present similar components. All components obtained in children presented an homologous component in young adults, although the order of components in children and young adults obtained by the amount of explained variance was different. These homologies between children and young adults, which are also present in similar PS topographies, implicate that the structure of the EEG is already present in children in the pre-adolescent period. Different components were extracted corresponding to beta-EMG, delta-theta, a temporo-occipital high alpha, occipital alpha, parieto-occipital low alpha, low delta and mu rhythm. Beta rhythm, given the extension to lateral sites would have an important contamination from Electromyography (EMG) in these lateral sites. These extracted rhythms broadly correspond to the frequency ranges which present a certain asynchrony in the maturational pattern.

It is remarkable that peaks of loading factors in children occur at lower frequencies than adults in most cases, and this is particularly clear in the three different sub-components of alpha and in the mu rhythm. The topographic representation of PS and component scores comparing homologous components between two age groups showed very similar topographies, despite the fact that the same frequencies were not represented for the PS of children and young adults.

When loading factors of the different alpha range sub-components of children and young adults are represented, a clear same displacement from lower to higher frequencies occurs from children to young adults. The scalp frequency shift to high frequencies during maturation has been proposed to be due to an increase in the contribution of high alpha to the alpha peak [32]. On the other hand, the presence of several components associated to alpha has been previously described by Tenke and Kayser [15] using power EEG, log power and amplitude spectra for extracting the PCA, and would represent different independent generators in alpha range. Therefore, the four alpha sub-components replicate the increase in frequency by maturation, which was also obtained in the scalp alpha rhythm in present report (Table 1), and which constitutes a central landmark of EEG development [18,31], and suggests that in addition to the high alpha contribution to the peak frequency shifting of alpha, a more extended shifting to higher frequencies occurs across the different alpha sub-components. However, Cragg et al. [32] suggested that the shifting in peak frequency is due to a greater contribution of alpha 2. This is possibly true for the age group analyzed by these authors (10–13 years old) but not for our group (peri-adolescent period), given that components scores of the high alpha and mu rhythms were not different in children and young adults (Table 2). Therefore, in our group of age, the increase of frequency in the alpha peak seems to occur for the acceleration of all the four sub-components in the alpha range with age.

A final point which deserves some comments is the previously obtained posterior extension of theta rhythm in children with respect to young adults. It has been proposed that theta band shows a maturational progression from posterior to anterior areas [9,18]. Theta activity has been described as located in posterior areas in children and could be explained as a precursor of the alpha rhythm in adults [9,10]. These previous studies used a broad band approach. The present narrow-band analysis showed that a parieto-occipital low alpha appears in both young adults and children. Therefore, it can be proposed from present analysis the existence of an independent parieto-occipital low alpha rhythm, peaking at lower frequencies in children than in young adults, which would be the cause of the apparent parieto-occipital extension of the theta rhythm in children [9,10].

The present results clearly show that the EEG structure is already present in late childhood, although with a higher PS and lower frequency than young adults. The maturation occurs in an asynchronic manner for different frequencies and locations.

Some important limitations of present report are (i) that present study is only able to capture differences between the different age groups and not inside a certain age group, in fact, a continuous recording for all possible age groups would provide a better description of EEG maturation, (ii) the cohort character of present study, which only measures differences between age groups, which is an indirect measure of maturation. However the normal population character of the subjects in this experiment suggests that the obtained results would have a strong relationship with normal EEG developmental maturation in the peri-adolescent period, and (iii), given the circadian and homeostatic pattern of changes in beta, alpha and theta rhythms [65], and the lack of control of the recording time, part of the obtained differences could be due to differences in recording time or sleep history between age groups.

## Conclusions

In this study we conclude the following:

→ Children have a higher absolute power than young adults for frequency ranges between 0–20 Hz. For relative PS children present a higher power than young adults in delta and theta range, while young adults have an increased spectral power in very low range (0 to 0.51 Hz) and beta frequencies.

→ The age comparisons of mean PS, the correlation of PS with age and the variance age comparison showed that there are six ranges of frequencies that can distinguish the level of EEG maturation in children and adults. Frequencies are: very low delta, delta- theta, low alpha, high alpha, low beta and high beta.

→ Both PS: absolute and relative showed good correlations with age, indicating that PS can be regarded as a maturation index of the human electroencephalogram. But they presented different topographies, indicating that some caution must be taken when comparing absolute and relative power.

→The age group mean comparisons of PS and the PS correlation with age suggest a postero-anterior maturation of the PS with the following order: High alpha, followed by low alpha and high beta, low beta and low delta and finally the delta-theta range.

→ Cross frequency correlation matrices suggest an asynchrony in the maturation of the different brain rhythms around peri-adolescent period.

→ The component 1 of non-rotated PCA would explain the individual variability.

→The representation of loading factors of different frequencies suggests that the increase in frequency with maturation occurs in most of the frequency ranges, and particularly in the alpha sub-components and mu rhythm.

→The component scores and PS topographies show very similar topographies in young adults and children, reinforcing the idea of a completed structure of the EEG rhythms in the pre-adolescent period. Interestingly, in the alpha range, a parietal low alpha, a posterior alpha, an occipito-temporal high alpha and a mu rhythm can be distinguished in the 7–13 Hz range.

## Abbreviations

(PS): Power Spectrum; (EEG): Electroencephalogram; (PCA): Principal Component Analysis; (SD): Standard Deviation; (KÎÂ©): Kilo-Ohms; (DC): Direct Current; (AD): Analog Digital; (ERPs): Even Related Potential; (BESA): Brain Electrical Source Analysis; (μV): Microvolts; (FFT): Fast Fourier Transform; (Hz): Hertz; (SPSS): Statistical Package for the Social Sciences; (ANOVA): **AN**alysis **O**f **VA**riance; (PRfi): Relative Power for frequency i; (PAfi): Absolute Power for frequency i; (N): Number; (EMG): Electromyography.

## Competing interests

'The authors declare that they have no competing interests'.

## Authors’ contributions

EI: Data Analysis and rational of the study. CI: Data Analysis and rational of the study. MI: Data Analysis and rational of the study. C: Data Analysis and rational of the study. AM: Data Analysis. CM: Data recording, Analysis, and rational of the study. All authors read and approved the final manuscript.

## Authors’ information

EI: Degree in Psychology. PhD student. CI: Degree in Psychology. PhD student. MI: Degree in Psychology. PhD student. C: Degree in Psychology. PhD student. AL: Professor of methodology of Behavior Sciences, Phd. CM: Professor of Psychobiology Phd.
